# The striking and unexpected cytogenetic diversity of genus *Tanacetum* L. (Asteraceae): a cytometric and fluorescent *in situ* hybridisation study of Iranian taxa

**DOI:** 10.1186/s12870-015-0564-8

**Published:** 2015-07-08

**Authors:** Nayyereh Olanj, Teresa Garnatje, Ali Sonboli, Joan Vallès, Sònia Garcia

**Affiliations:** Department of Biology, Faculty of Basic Science, Malayer University, Malayer, Iran; Laboratori de Botànica – Unitat associada CSIC, Facultat de Farmàcia, Universitat de Barcelona, Avinguda Joan XXIII s/n, 08028 Barcelona, Catalonia Spain; Institut Botànic de Barcelona (IBB-CSIC-ICUB), Passeig del Migdia s/n, Parc de Montjuïc, 08038 Barcelona, Catalonia Spain; Department of Biology, Medicinal Plants and Drugs Research Institute, Shahid Beheshti University, Evin, 1983963113 Tehran Iran

**Keywords:** 5S, 35S, Aneuploidy, Evolutionary cytogenetics, Genomic instability, L-type arrangement, Polyploidy, Odd ploidy, Ribosomal DNA

## Abstract

**Background:**

Although karyologically well studied, the genus *Tanacetum* (Asteraceae) is poorly known from the perspective of molecular cytogenetics. The prevalence of polyploidy, including odd ploidy warranted an extensive cytogenetic study. We studied several species native to Iran, one of the most important centres of diversity of the genus. We aimed to characterise *Tanacetum* genomes through fluorochrome banding, fluorescent *in situ* hybridisation (FISH) of rRNA genes and the assessment of genome size by flow cytometry. We appraise the effect of polyploidy and evaluate the existence of intraspecific variation based on the number and distribution of GC-rich bands and rDNA loci. Finally, we infer ancestral genome size and other cytogenetic traits considering phylogenetic relationships within the genus.

**Results:**

We report first genome size (2C) estimates ranging from 3.84 to 24.87 pg representing about 11 % of those recognised for the genus. We found striking cytogenetic diversity both in the number of GC-rich bands and rDNA loci. There is variation even at the population level and some species have undergone massive heterochromatic or rDNA amplification. Certain morphometric data, such as pollen size or inflorescence architecture, bear some relationship with genome size. Reconstruction of ancestral genome size, number of CMA+ bands and number of rDNA loci show that ups and downs have occurred during the evolution of these traits, although genome size has mostly increased and the number of CMA+ bands and rDNA loci have decreased in present-day taxa compared with ancestral values.

**Conclusions:**

*Tanacetum* genomes are highly unstable in the number of GC-rich bands and rDNA loci, although some patterns can be established at the diploid and tetraploid levels. In particular, aneuploid taxa and some odd ploidy species show greater cytogenetic instability than the rest of the genus. We have also confirmed a linked rDNA arrangement for all the studied *Tanacetum* species. The labile scenario found in *Tanacetum* proves that some cytogenetic features previously regarded as relatively constant, or even diagnostic, can display high variability, which is better interpreted within a phylogenetic context.

**Electronic supplementary material:**

The online version of this article (doi:10.1186/s12870-015-0564-8) contains supplementary material, which is available to authorized users.

## Background

*Tanacetum* L. is a genus of the family Asteraceae Bercht. & J. Presl and includes approximately 160 species [1]. It is one of the largest genera within the tribe Anthemideae Cass., together with genera such as *Artemisia* L., *Achillea* L. and *Anthemis* L. Commonly known as tansies, *Tanacetum* species are native to many areas of the Northern Hemisphere, occupying the temperate zones of Europe, Asia, North Africa and North America, but particularly abundant in the Mediterranean and Irano-Turanian regions. Although the presence of *Tanacetum* in the Southern Hemisphere is much less common [1, 2], some species are grown worldwide such as *T. parthenium* (L.) Sch. Bip., which can behave as a weed outside its native range.

*Tanacetum* species are mostly perennial herbs, although the genus has some annuals and some subshrubs. They usually form rhizomes and are aromatic plants. Their capitula, solitary or arranged in more or less dense or loose compound inflorescences, always contain disc flowers (flosculous, yellow, numerous — up to 300), sometimes with ray flowers (ligulate, white, yellow or pale pink). *Tanacetum* is considered to hold a crucial position for understanding the phylogenetic relationships within its tribe, but available phylogenetic reconstructions show that these species form an imbroglio whose generic relationships and infrageneric arrangement are still unsettled [3]. Many species of *Tanacetum* are widely distributed and are used as sources of medicines, food or forage. In particular, several studies have shown that essential oils from *T. parthenium* [4–6] and *T. balsamita* L. [7–9] have strong antibacterial, cytotoxic, neuroprotective and antioxidant activity. *T. balsamita* has also shown anti-inflammatory properties [10]. West and central Asia are two important speciation centres of the genus [11], and Iran is one of the main areas of speciation and diversification, promoted by a unique combination of ecosystems. In Iran the genus is represented by 36 species according to the most recent revisions, including 16 endemic taxa [3, 12–17].

The karyology of *Tanacetum* has been extensively studied, with chromosome counts known for a considerable number of its species [18–21]. Its basic chromosome number is x = 9, as in most Anthemideae and Asteraceae; indeed x = 9 is likely the ancestral basic number for the family as a whole [22]. Ploidy levels are found up to 10× [23]. Recent work has added more karyological information for this genus; it seems that polyploidy is an important evolutionary force and the existence of odd ploidy, aneuploidy and presence of B-chromosomes is not uncommon [18, 20].

Methods such as fluorochrome banding and fluorescent *in situ* hybridisation (FISH) of 5S and 18S-5.8S-26S (35S) ribosomal RNA genes (rDNA) provide chromosome markers, excellent tools to improve karyotype description [24]. These methods have proven useful for comparing taxa at different levels, particularly in plants (see, for example, [25] on several Asteraceae genera; [26], on *Fragaria* L.; [27] on *Thinopyrum* Á. Löve). However broader cytogenetic information is largely missing for *Tanacetum*, as happens for many wild species, unlike crops or other economically interesting plants whose chromosomes have been more deeply investigated. Genome size estimation, easily obtained by flow cytometry, has been used in a similar way (see, for example, [28] on *Tripleurospermum* Sch. Bip.; [29] on *Carthamus* L.; [30] on *Artemisia* L.). The combination of these methods can improve our understanding of chromosome evolution and genome organisation processes in plants [31]. Moreover, molecular cytogenetic studies, together with genome size evaluation, are also useful in a wide range of biological fields, from taxonomy, evaluation and conservation of genetic resources, to plant breading [24, 32–34].

Despite being a large and well-known genus, molecular cytogenetic studies of *Tanacetum* are limited to a single work reporting data on two species: *T. achilleifolium* (M. Bieb.) Sch. Bip. and *T. parthenium* [35]. That study described co-localisation of both 5S and 35S ribosomal RNA genes in *Tanacetum*, the so-called linked type (L-type) arrangement of rDNA, confirming preliminary findings for this genus [25]. This rDNA organisation is typical of several Asteraceae members, particularly those belonging to tribes Anthemideae and the Heliantheae Cass. alliance (see [25, 36] for details). However, the most common rDNA organisation in plants, and also in family Asteraceae, is that in which both rRNA genes are separated (S-type arrangement). Remarkably, [35] found that one 35S rDNA locus was separated in *T. achilleifolium*, while the other one remained co-localised with the 5S. This dual organisation of rDNA in the same species (i.e. both L-type and S-type coexisting) is exceptional.

Likewise, genome sizes for *Tanacetum* are only known for few species, reduced to three research works to our knowledge [37–39]. In this study, we establish a deeper knowledge of *Tanacetum* genomes through molecular cytogenetic and genome size analysis. We focus on several species native to Iran, since this area constitutes a centre of speciation and diversification of the genus. All ploidy levels previously reported for the genus (from 2x to 10x) exist in Iran [20], many of the studied tansies grow there in polyploid series, and odd stable ploidy, aneuploidy and presence of B-chromosomes have been found [3, 20]. Our specific goals were (1) to characterise the genomes of *Tanacetum* species by flow cytometry, fluorochrome banding and FISH of rRNA genes, and particularly, to observe the rDNA organisation in these species, (2) to detect the karyotype and genome size patterns of the genus and describe their typical models, if any, (3) to address the presence of polymorphisms at the cytogenetic level, (4) to assess the impact of polyploidy in *Tanacetum* genomes, and (5) to reconstruct ancestral character states of genome size and karyotype features such as number of rDNA loci and CMA+ bands to infer genome evolution in the context of a phylogenetic framework of the genus.

## Results

The chromosome counts here represent most ploidy levels found in *Tanacetum* to present, all x = 9-based. We found B-chromosomes in one of the populations of *T. pinnatum* and in *T. fisherae*, and some of the populations investigated, such as those of *T. archibaldii* and *T. aureum* (Lam.) Greuter, M.V.Agab. & Wagenitz, presented mixed ploidy. In addition, several of the studied taxa are odd polyploids, such as the case of triploid *T. joharchii* Sonboli & Kaz. Osaloo and *T. kotschyi* (Boiss.) Grierson, and the pentaploid *T. fisherae* Aitch. & Hemsl. which is also a hypoaneuploid since it has lost one chromosome out of the 45 expected. More detailed karyological information is in Table 1.

**Table 1:** Provenance and voucher number from the Medicinal Plants and Drug Research Institute Herbarium (MPH), Shahid Beheshti University (Tehran) of the populations of *Tanacetum* studied, together with genome size, number of CMA+ bands and number of rDNA sites

Species	Population	PL^1^	2n^2^	2C^3^	2C^4^	SD^5^	1Cx^6^	HPCV^7^	CMA^8^	rDNA^9^
*T. archibaldii* Podl.	Mazandaran: Pole Zangoleh road (1790)	2	18	8.77	8577	0.04	4.39	1.77	56itc (50, 54, 66)	4
*T. balsamita* L.	Mazandaran: Pole Zangooleh road (1788)	2	18	10.38	10152	0.09	5.19	1.13	40tc (24, 30, 34, 36, 40, 42, 44)	4
*T. budjnurdense* (Rech.f) Tzvel.	Khorasan: Bujnourd (1477)	2	18	10.13	9907	0.19	5.07	1.77	4t	4
*T. canescens* DC.	Zanjan: Soltanieh (1912)	2	18	9.3	9095	0.13	4.65	1.68	4, 6 and 8tc	6 (8)
*T. aureum* (Lam.) Greuter, M.V.Agab. & Wagenitz	Urmia: Meyab (1848)	4	36	17.08*	16704	1.38	4.27	2.62	28tc (26, 32, 34)	10 (8)
*T. aureum* (Lam.) Greuter, M.V.Agab. & Wagenitz	Urmia: Suluk Waterfall (1861)	4	36	15.47*	15130	0.36	3.87	2.79	6 and 10t (3, 4, 5)	10
*T. heimerlii* (Nabělek) Parsa	Urmia: Sero road, Golsheykhan (1227)	2	18	8.25	8069	0.06	4.13	2.09	4t (2, 3, 5, 6)	4 and 6
*T. oligocephalum* (DC.) Sch.Bip.	Urmia: Chaldoran (1914)	2	18	7.67	7501	0.05	3.84	2.53	6t (4)	6
*T. oligocephalum* (DC.) Sch.Bip.	Urmia: Naghadeh (1868)	4	36	17.57*	17183	0.62	4.39	2.2	22t(10, 12, 14, 20, 24)	12 (8, 10)
*T. oligocephalum* (DC.) Sch.Bip.	Urmia: Mamakan (1911)	4	36	14.87*	14543	0.28	3.72	3.02	10t (8, 9)	10
*T. fisherae* Aitch. & Hemsley.	Kerman Mehr mountain, north and east slopes (1916)	5	44^A^	17.11*	16734	0.27	3.42	2.69	30 tc (8, 14, 22, 24, 28)	10 (5, 7, 6, 12, 15)
*T. hololeucum* (Bornm.) Podl.	Mazandaran: Pole Zangoleh road (1791)	2	18	8.45	8264	0.2	4.23	1.61	14 and 16t (18, 20, 22)	6
*T. joharchii* Sonboli & Kaz.Osaloo	Khorasan, Chenaran, (1620)	3	27	11.31*	11061	0.11	3.77	0.92	24itc (32 and 36)	6 (5, 8)
*T. kotschyi* (Boiss.) Grierson	Urmia, Anhar road, Suluk (1129)	3	27	10.04*	9819	0.07	3.35	1.63	24tc (20, 28, 32, 34)	6
*T. kotschyi* (Boiss.) Grierson	Tabriz: Mishodagh (1339)	3	27	10.72*	10484	0.12	3.57	1.83	44tc (28, 32, 42, 44, 48)	6
*T. kotschyi* (Boiss.) Grierson	Zanjan: Ghidar (1419)	3	27	8.58*	8391	0.09	2.86	1.89	18tc (20, 22, 26)	4
*T. parthenifolium* (Willd.) Sch.Bip.	Urmia: Suluk Waterfall (1127)	2	18	4.68	4577	0.09	2.34	3.07	4t	4
*T. parthenium* (L.) Sch.Bip.	Tehran: Tochal (1483)	2	18	3.84	3756	0.04	1.92	2.46	2t (3, 4)	2 (3, 4)
*T. parthenium* (L.) Sch.Bip.	Tehran: Shahid Beheshti University, agricultural field of research. Cultivated (1633)	2	18	4.51	4411	0.04	2.26	3.06	14tc (8, 10)	6
*T. parthenium* (L.) Sch.Bip.	Hamadan: Dare Morad Beig (1903)	2	18	4	3912	0.04	2.00	3.02	3t (2,4)	2(3, 4)
*T. persicum* (Boiss.) Mozaff.	Chahar Mahal & Bakhtiari: Sabz Kuh (1502)	2	18	4.4	4303	0.69	2.20	2.49	4t	4
*T. pinnatum* Boiss.	Hamadan: Asad Abad (1895)	2	18^B^	13.19	12900	0.06	6.60	2.09	4t	4
*T. pinnatum* Boiss.	Hamadan: Malayer (1896)	2	18	13.18*	12890	0.08	6.59	2.75	4t (6)	4
*T. pinnatum* Boiss.	Hamadan: Razan (1894)	4	36	24.87*	24323	0.58	4.15	1.45	6t (3, 4, 5)	4 and 6 (8)
*T. polycephalum* Sch.Bip. ssp. *argyrophyllum* (K.Koch) Podlech	Urmia: Meshkin Shahr (1884)	2	18	9.26	9056	0.14	4.63	1.3	6t (5, 7, 8, 10)	6 (7, 8)
*T. polycephalum* Sch.Bip. ssp. *argyrophyllum* (K.Koch) Podlech	Urmia: Ghasemloo Valley (1866)	4	36	17.88*	17487	0.84	4.47	2.84	8 and 10t (5, 6, 13)	12 (14)
Species	Population	PL^1^	2n^2^	2C^3^	2C^4^	SD^5^	1Cx^6^	HPCV^7^	CMA^8^	rDNA^9^
*T. polycephalum* Sch.Bip. ssp. *argyrophyllum* (K.Koch) Podlech	Urmia: Oshnaviyeh (1867)	4	35	16.82*	16450	0.4	4.21	2.94	6, 10, 20 and 24t	14 (10, 11, 12, 13, 15)
*T. polycephalum* Sch.Bip. ssp. *argyrophyllum* (K.Koch) Podlech	Urmia: Marand (1856)	4	36	17.89*	17496	0.16	4.47	2.4	32 and 36t (8, 20)	12 (14)
*T. polycephalum* Sch.Bip.ssp. *azerbaijanicum* Podlech	Urmia: Ghishchi (1212)	4	36	18.24*	17839	0.31	4.56	2.4	16t (8, 14)	12
*T. polycephalum* Sch.Bip. ssp. *duderanum* (Boiss.) Podlech	Mazandaran: Pole Zangoleh road (1795)	4	36	17.63*	17242	0.53	4.41	3.22	14tc (18, 20, 22, 24)	12 (11)
*T. polycephalum* Sch.Bip. ssp*. farsicum* Podlech	Hamadan: Kabudar Ahang (1901)	6	54	24.12**	23589	0.39	4.02	3.46	22 and 24t (18, 20, 26)	13 (14, 17)
*T. polycephalum* Sch.Bip. ssp*. heterophyllum* (Boiss.) Podlech	Mazandaran: Pole Zangoleh road (1797)	4	36	18.10*	17702	0.29	4.53	2.48	18 and 22t (16, 18, 20, 30, 32)	12 (9, 10, 11)
*T. polycephalum* Sch.Bip.ssp. *heterophyllum* (Boiss.) Podlech	Hamadan: Asad Abad (1899)	6	54	22.99**	22484	0.56	3.83	2.88	8t (10, 12, 14, 16)	18 (15, 16, 17)
*T. sonbolii* Mozaff.	(305) Urmia: Takab	2	18	9.17	8968	0.19	4.59	2.12	5t (4, 6, 8)	8
*T. tabrisianum* (Boiss.) Sosn. & Takht.	Urmia: Ahar (1905)	6	54	23.56**	23042	1.12	3.93	2.59	20 and 26t (14, 16, 27)	14 and 16 (10, 12)
*T. tabrisianum* (Boiss.) Sosn. & Takht.	Urmia: Ahar (1906)	6	54	24.01**	23482	0.16	4.00	1.96	50t (28, 40)	16 (14, 15, 26)
*T. tenuisectum* (Boiss.) Podl.	Tehran: Damavand (863)	2	18	7.68	7511	0.13	3.84	1.11	32, 34 and 46tc	6 (8, 10)
*T. tenuissimum* (Trautv.) Grossh.	Urmia: Jolfa (1855)	4	36	16.26*	15902	1.33	4.07	2.74	16 and 22 tc	9

All populations are native to Iran. (1) ploidy; (2) chromosome number; (3) genome size in pg; *Petunia hybrida* ‘PxPC6’ (2C = 2.85 pg), (*) *Pisum sativum* ‘Express Long’ (2C = 8.37 pg), and (**) *Triticum aestivum* ‘Chinese Spring’ (2C = 30.9 pg) were used as internal standards; (4) genome size in Mbp (1 pg = 978 Mbp); (5) standard deviation; (6) monoploid genome size; (7) half peak coefficient of variation for each population; (8) most commonly found number of CMA+ bands, together with the most usual position found for them (I = interstitial, t = terminal or subterminal, c = centromeric or pericentromeric); in brackets, other numbers of CMA+ bands found; (9) most commonly found number of rDNA sites; in brackets other numbers of rDNA sites found (position of rDNA sites is always terminal or subterminal). ^A^The expected number for a pentaploid would be 2n = 45 but there is an already described hypoaneuploidy for this taxon, sometimes presenting a B chromosome (2n = 44 + 1B, [105]); ^B^two to three B-chromosomes occasionally found

### Genome size

**Fig. 1:**
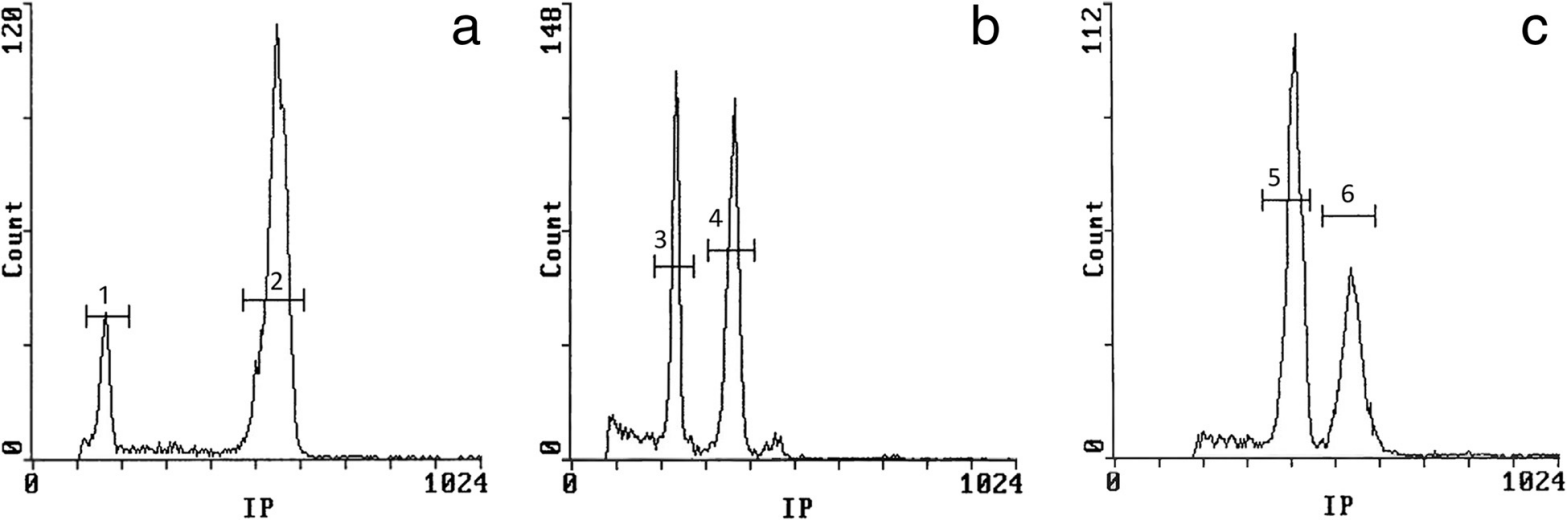
Fluorescence histograms of the genome size assessments of (**a**) *T. heimerlii* 2x population (2) with *Petunia hybrida* (1) as internal standard, (**b**) *T. pinnatum* 4x population (4) with *Pisum sativum* (3) as internal standard and (**c**) *T. polycephalum* ssp. *heterophyllum* 6x population (5) with *Triticum aestivum* (6) as internal standard

Table 1 presents holoploid genome size data (2C), together with other karyological features of the studied species, as well as information on some closely related taxa for comparison. We analysed 38 populations of 20 species and five subspecies of *Tanacetum*, including ploidy from 2x to 6x. Genome sizes (2C) ranged from 3.84 pg (belonging to one of the diploid populations of *T. parthenium*) to 24.87 pg (from a tetraploid population of *T. pinnatum* Boiss.), an overall 6.47-fold range, and a 3.29-fold range at the diploid level. Mean 2C at diploid level is 8.05 pg. The low Half Peak Coefficient of Variation (HPCV) mean value (2.29 %) indicates good quality of the flow cytometric assessments. Fluorescence histograms from the flow cytometer are presented in Fig. 1 to illustrate the accuracy of measurements with all internal standards used.

We found intraspecific genome size differences in most cases in which several populations were assessed, reaching 22.18 % in the triploid *T. kotschyi*, 16.04 % in the diploid *T. parthenium*, 9.43 % in the tetraploid populations of *T. aureum*, 8.10 % in the tetraploid *T. polycephalum* Sch. Bip., 1.89 % in the hexaploid *T. tabrisianum* (Boiss.) Sons. & Takht., and negligible variability (<0.1 %) among diploid T*. pinnatum* populations*.*

Genome size (2C) and total karyotype length (TKL) were significantly (*p* < 0.0001) and positively correlated with ploidy, but monoploid genome size (1Cx) did not decrease with ploidy. Nevertheless, when data of the same species at different ploidy levels was compared, there was a trend to genome downsizing i.e. reduction of monoploid genome size in *T. polycephalum* and *T. pinnatum*, whose 4× and 6× polyploids present, respectively, 6.07 % and 17.96 % less genome size than expected from the genome size in their diploid populations. In addition, genome size is positively correlated with TKL (*p* = 0.003), with the number of rDNA signals (*p* < 0.0001) and with pollen morphometric characters such as polar axis (*p* = 0.03) and equatorial diameter (*p* = 0.02). Species with different compound inflorescences have significantly different genome sizes (*p* = 0.009); species with solitary capitula have the smallest genome compared to species presenting corymbs of capitula, which have the greatest amounts of DNA (5.54 pg vs 13.2 pg at the diploid level).

### GC-rich regions

**Fig. 2:**
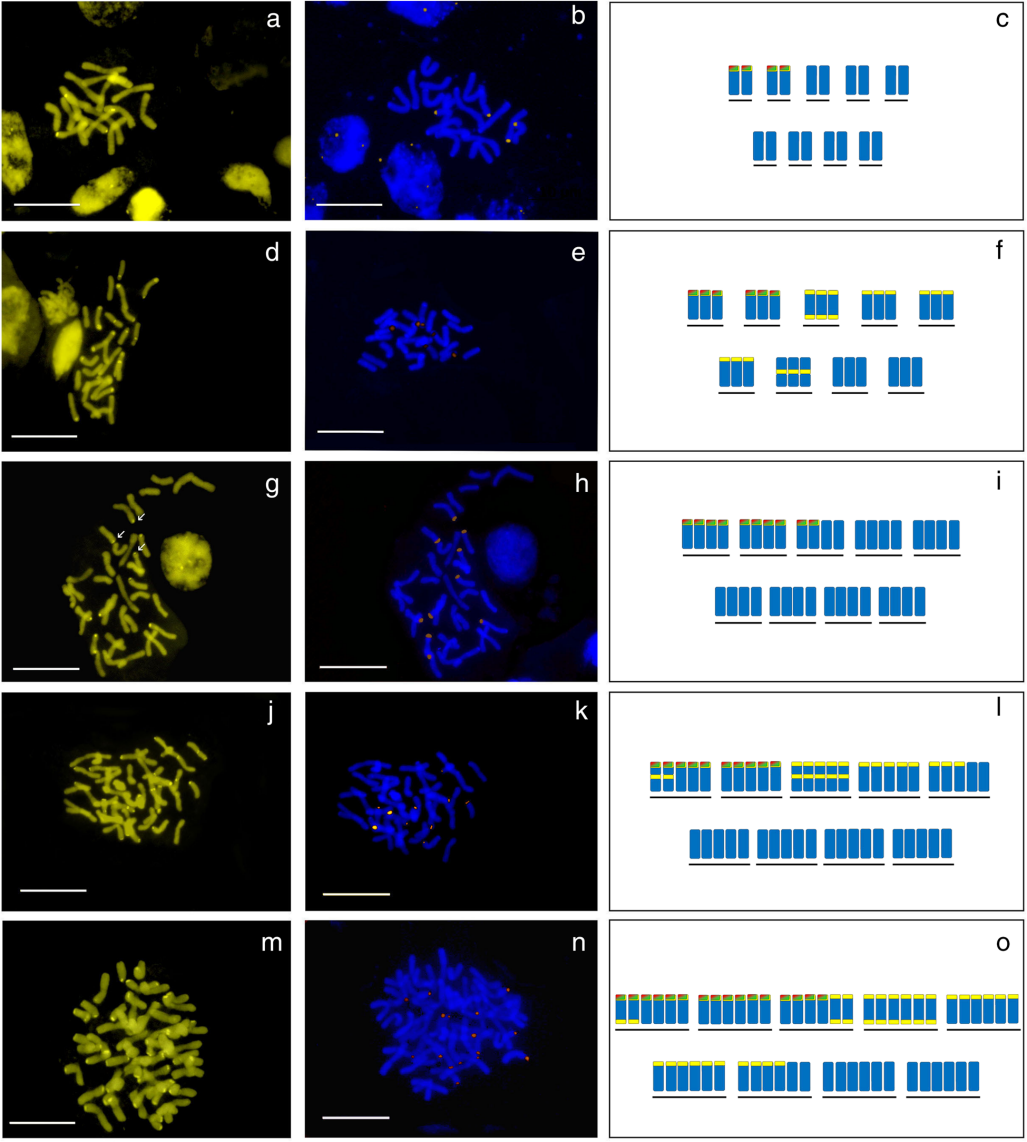
Chromomycin A_3_-positive (CMA+) and FISH images of the most commonly found metaphases of representative species of each ploidy level in *Tanacetum*. CMA+ bands are marked yellow, 26S-5S rDNA signals, marked orange in images. CMA+ positive bands are marked yellow, 26S-5S rDNA signals, are marked red-green in the schematic representation of chromosomes. (**a**, **b**, **c**) *Tanacetum pinnatum*, 2x population (Asad Abad, 1895) showing four CMA+ and four rDNA signals; (**d**, **e**, **f**) *T. kotschyii*, 3x population (Urmia, 1129) showing up to 24 CMA and six rDNA signals; large CMA+ bands indicated with asterisks; (**g**, **h**, **i**) *T. oligocephalum*, 4x population (Mamakan, 1911), showing 10 CMA+ and 10 rDNA signals; large CMA+ bands indicated with asterisks and faint bands indicated with arrows; (**j**, **k**, **l**) *T. fisherae*, 5x population, showing up to 30 CMA+ and 10 rDNA signals; large rDNA signals indicated with asterisks; (**m**, **n**, **o**) *T. tabrisianum* 6x population (Ahar, 1906), showing up to 50 CMA+ and 16 rDNA signals; large rDNA signals indicated with asterisks. Scale bars = 10 μm

**Fig. 3:**
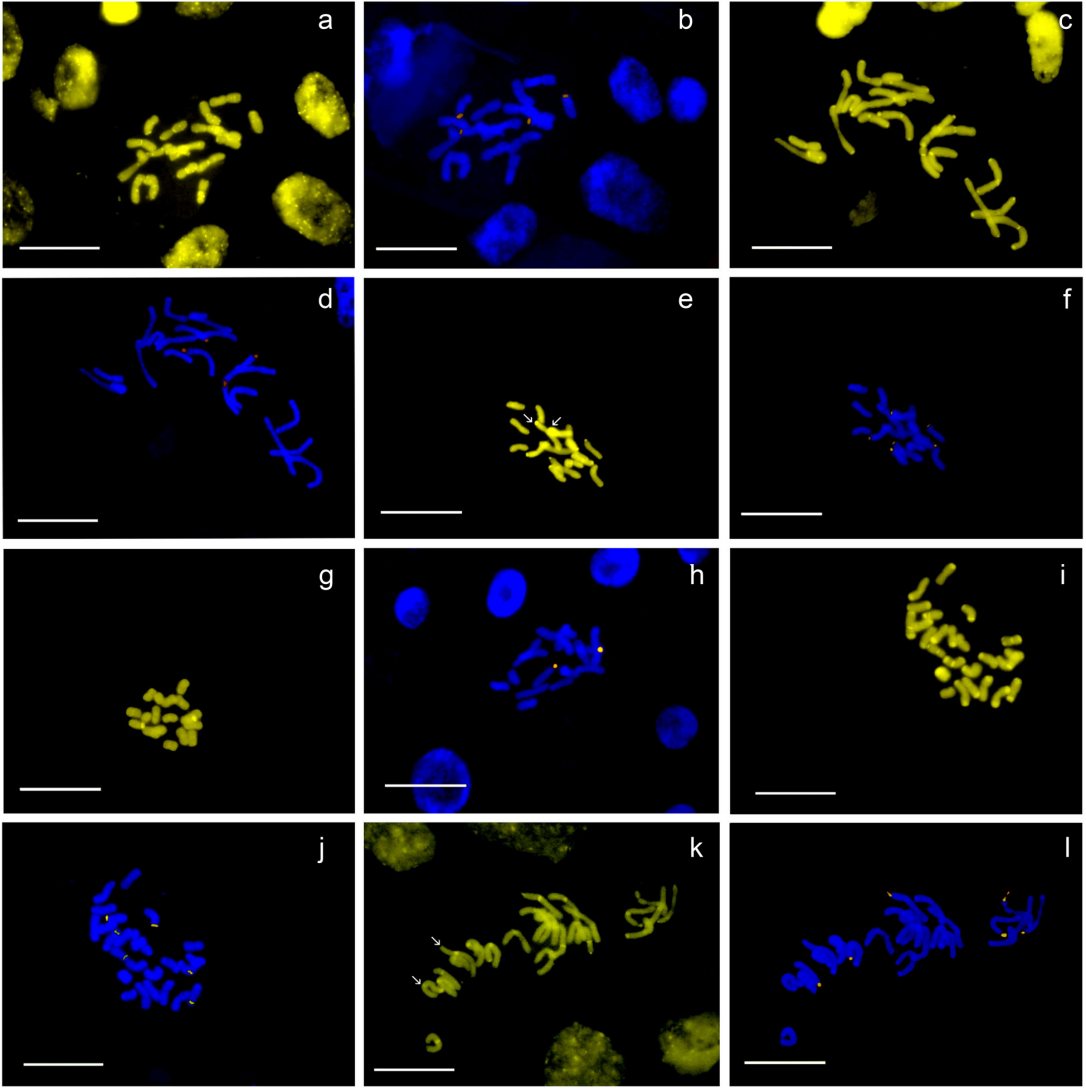
Chromomycin A_3_-positive (CMA+) FISH images of cytogenetically variable *Tanacetum* species, in which CMA+ bands are marked yellow, 26S-5S rDNA signals and marked orange. (**a**, **b**) *T. archibaldii* (2x) with 56 CMA signals (asterisks indicate interacalary CMA+ bands) and with 4 rDNA signals; (**c**, **d**) *T. balsamita*, 2x, with 40 CMA+ signals (many of them pericentromeric, indicated with asterisks) and with four rDNA signals – a slightly decondensed rDNA is indicated with an arrow; cultivated (**e**, **f**) and wild (**g**,**h**) *T. parthenium* (from Shahid Beheshti University, 1633 and Tochal, 1483, respectively), both 2x with 14 and six CMA+ and six and two rDNA signals observed, respectively; (**i**, **j**) *T. kotschyi* (Tabriz, Mishodagh, 1339), 3x, with 44 CMA+ signals and six rDNA signals and (**k**, **l**) *T. joharchii*, 3x, with 24 CMA and six rDNA signals; note faint or interstitial CMA+ bands indicated with asterisks and decondensed rDNAs indicated with arrows in both pictures. Scale bars = 10 μm

Table 1 shows the results of fluorochrome banding with chromomycin and FISH assays, and Figs. 2 and 3 present selected representative *Tanacetum* metaphases. For the sake of clarity, only three chromosomal locations have been considered both for chromomycin A_3_ (CMA) and rDNA signals, following the treatment used in the www.plantrdnadatabase.com. These are: (peri)centromeric, interstitial and (sub)terminal. Results of chromomycin banding, which stains GC-rich DNA portions, are highly variable within and between *Tanacetum* species and even among individuals in some cases. In only four species is the number of bands always constant (the diploids *T. parthenifolium* Sch. Bip., *T. persicum* (Boiss.) Mozaff., *T. pinnatum* and *T. budjnurdense* (Rech.f.) Tzvelev) and low — four, see picture of *T. pinnatum* (Fig. 2a). However, from a minimum of two CMA+ bands in a wild population of the diploid *T. parthenium* (Fig. 3g) to a maximum of 66 bands for the diploid *T. archibaldii* Podlech (Fig. 3a) there are myriad variations. In most cases, however, there is a considerable range of variability within a species. The preferred position is usually (sub)terminal, and sometimes detached or terminal decondensed DNA (probably rDNA) is clearly seen with this staining (see Fig. 3k). Several species also present pericentromeric bands, and in two species (*T. archibaldii* and *T. joharchii*), several intercalary signals are also visible (Fig. 3a and 3k). Pericentromeric (and to a lesser extent, intercalary) bands appear in species that already present a high number of GC-rich bands.

Several taxa of different ploidy (different populations from *T. aureum, T. heimerlii* (Nábělek) Farsa*, T. parthenium, T. polycephalum* Sch. Bip. subsp. *argyrophyllum* (K.Koch) Podlech*, T. pinnatum, T. sonbolii* Mozaff. and *T. tabrisianum*) show odd numbers of bands in different individuals (Table 1). Intensity and size differences of chromomycin signals are clearly visible in several species, such as *T. kotschyi* (Fig. 2d), *T. oligocephalum* (DC.) Sch. Bip. (Fig. 2g), *T. balsamita* (Fig. 3c) and *T. joharchii* (Fig. 3k).

There is no significant relationship between ploidy and the most commonly found number of signals for a given species, nor with genome size. In addition, the number of GC-rich bands is positively correlated with the altitude at which species occur, considering all taxa (*p* = 0.04) and only diploids (*p* = 0.006).

### rDNA loci

The FISH assays of a large sample representing genus *Tanacetum* show a totally homogeneous L-type organisation of ribosomal RNA genes. The number of signals within a species (even within a population) and between species at the same ploidy is usually heterogeneous although not as heterogeneous as the number of CMA+ bands. The minimum number of signals found was two (one locus) for one population of *T. parthenium* and the maximum was 26 (13 loci) for some individuals of one population of *T. tabrisianum* (although most *T. tabrisianum* had eight loci, see Fig. 2n). In all cases, rDNA signals occupied terminal or subterminal positions, always coincidental with CMA+ signals, and sometimes appearing as decondensed (as *T. joharchii* in Fig. 3d, l arrows). Species such as *T. fisherae* and *T. tabrisianum* (Fig. 2k, n, asterisks), presented locus size differences, but in general, this was homogeneous. The number of rDNA signals was positively and significantly correlated with ploidy and genome size (*p* < 0.0001 for both), but there was no reduction in number of loci, as the number of signals per haploid genome did not diminish significantly with increasing ploidy. However, a reduction in the number of signals was detected in individual polyploid series for *T. pinnatum* and three out of four of *T. polycephalum*. In all other cases there was additivity; that is, the tetraploid had exactly twice as many signals as the diploid, except in the case of one tetraploid *T. polycephalum* population, in which there was upsizing by one locus.

The heterogeneity in the number of signals for a given species (that is, the different number of rDNA loci that could be found in metaphases coming from the same species) was positively correlated with ploidy (*p* < 0.0001) which means that with increasing ploidy there was a tendency to instability in the number of rDNA signals. In particular, the hypoaneuploid *T. fisherae* (2n = 5x = 44) and *T. polycephalum* var. *argyrophyllum* (2n = 4x = 35) were the most unstable with respect to the number of rDNA signals.

### Phylogenetic relationships among species and ancestral characters

**Fig. 4:**
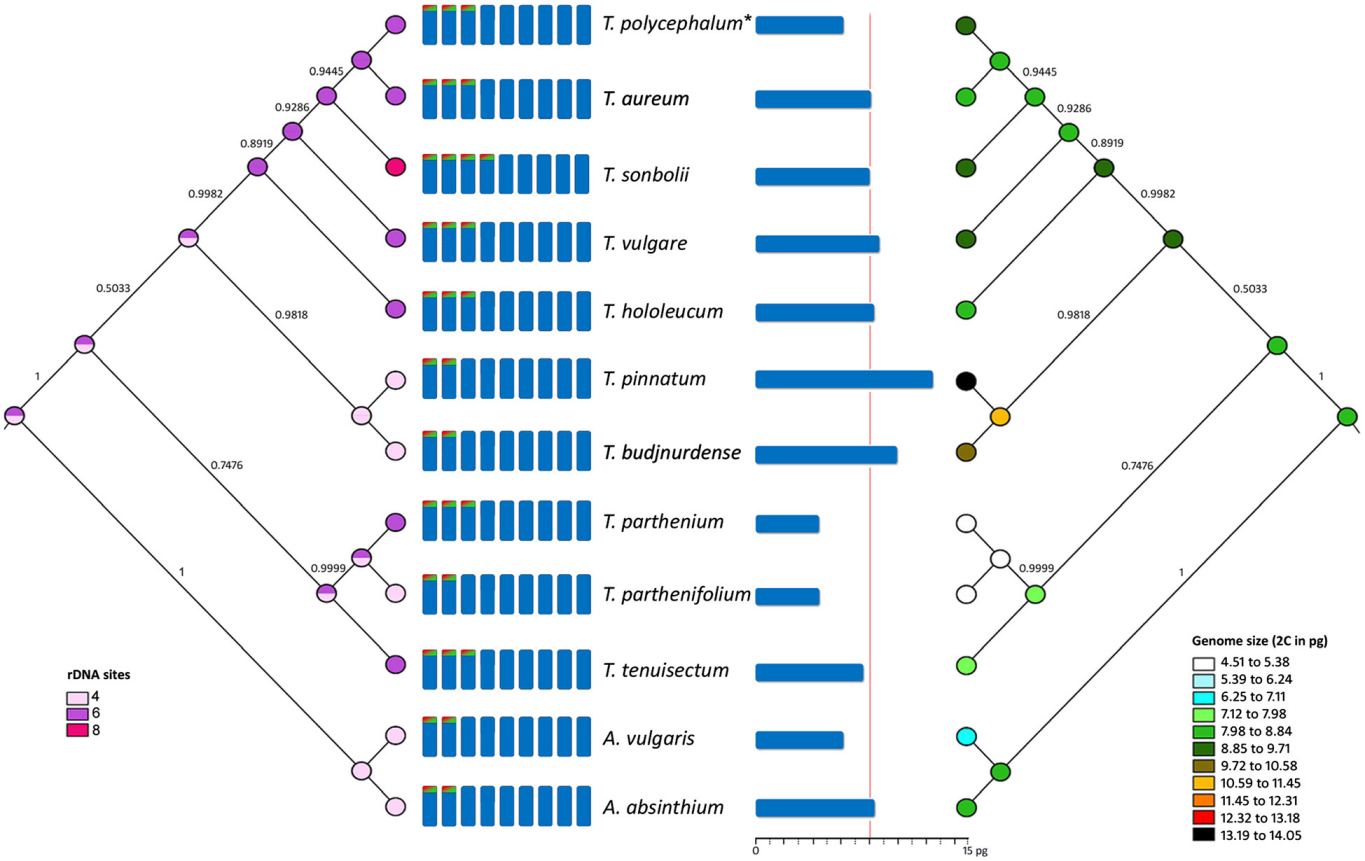
Ancestral state reconstruction of number of rDNA signals (left) and genome size (right) for diploid *Tanacetum* taxa. The model of reconstruction was Parsimony as implemented in Mesquite (v.3.02), and ancestral state reconstruction was estimated using the 50 % majority-rule consensus topology obtained by Bayesian inference phylogenetic analysis of the internal transcribed spacer 1 (ITS1), ITS2 and *trn*H-*psb*A data sequence. The Bayesian clade-credibility values (posterior probability > 0.5) are given above branches. Schematic representation of chromosomes with the most commonly found numbers of rDNA signals and bars that depict genome sizes (2C values) with a red line indicating the mean 2C value at the diploid level. (*) *Tanacetum polycephalum* ssp. *argyrophyllum*

Statistical analyses at the genus level should consider phylogenetic relationships among taxa to be as unbiased as possible. However, due to lack of enough data, these comparisons could not be done in most cases. Still, we detected significant and positive correlations using the phylogenetic generalised least squares method (PGLS) between genome size (2C), ploidy, and number of rDNA signals (*p* < 0.0001), i.e. all parameters increase/decrease in concert. The reconstruction of character evolution into the phylogeny (Fig. 4), based on diploid taxa, provides ancestral 2C values ranging from 7.98 to 8.84 pg, from 10 to 13 for CMA+ bands, and from 4 to 6 rDNA signals for *Tanacetum* species.

## Discussion

All species investigated present x = 9 as the basic chromosome number confirming previous research [20, 23]. In contrast to other Anthemideae, in which other basic chromosome numbers have been found (e.g. *Artemisia* presents x = 7, 8, 9, 10, 11; *Pentzia* Thunb., x = 7, 8, 9, *Lasiospermum* Fisch., x = 9, 10 [40]) x = 9 it is the only found in *Tanacetum* until present [41].

To our knowledge, genome size was available for only four species of the genus, the diploid *T. vulgare* (mean 2C = 8.85 pg, [37]), a tetraploid population of *T. cinerariifolium* (Trevir.) Sch. Bip. (2C = 14.53 pg, [38]) and some hexaploid populations of *T. balsamita* and *T. corymbosum* (L.) Sch. Bip. (2C = 21.44 pg and 2C = 19.95 pg, respectively, [39]). Therefore this research contributes new genome sizes for all species and subspecies studied here (with the exception of *T. balsamita*), representing approximately 11 % of the recognised species of the genus. The amount of nuclear DNA is mostly intermediate in *Tanacetum*. According to the genome size categories in plants established by [42], three of the 20 species we studied (17.65 %) have small genome sizes (2.8 ≤ 2C < 7 pg), whereas the remaining have intermediate genome sizes (7 ≤ 2C < 28 pg), including all ploidy levels. Mean genome size of the diploid taxa studied (8.35 pg) was coincidental with the mean of the tribe Anthemideae (8.30 pg) and of the family Asteraceae (2C = 8.20 pg), according to data from the Genome Size in Asteraceae Database (www.asteraceaegenomesize.com). Closely related diploid genera, such as *Artemisia*, have similar mean genome sizes (2C = 7.75 pg) whereas the majority of diploid *Tanacetum* allies present remarkably lower mean 2C values (2C = 5.9 pg for *Achillea*, 2C = 6.4 pg for *Anacyclus* L., 2C = 5.12 for *Anthemis*, 2C = 5.71 for *Matricaria* L., 2C = 5.13 for *Tripleurospermum*). The comparatively larger mean genome size of *Tanacetum* could be because our sample lacks annual representatives (as does most of the genus) which, quite often — though not always — tend to present lower genome sizes than their counterparts [43].

### Genome downsizing and polyploidy in *Tanacetum*

Polyploidy and hybridisation are important evolutionary forces shaping plant genomes and underlying the huge angiosperm diversity. Both can confer evolutionary advantages [44–46] attributed to the plasticity of plant genomes and to increased genetic variability, generating individuals capable of exploiting new niches [47]. Polyploidy is linked to numerous epigenetic/genomic changes such as chromosome rearrangements, transposable element mobilisation, gene silencing or genome downsizing [48–50]. Certainly, genome downsizing would be a widespread biological response to polyploidisation [51]. This may lead to diploidisation of the polyploid genome [52–54]. There is no evidence of genome downsizing across *Tanacetum* ploidy levels. However, there are genome size trends within separately polyploid series of particular species. Tetraploid *T. pinnatum* presents up to 6.07 % lower 1Cx than expected from the 1Cx of the diploid populations, and hexaploid and tetraploid *T. polycephalum* present, respectively, 17.96 % and 4.28 % lower 1Cx than expected from the 1Cx of the diploid population. This is consistent with previous observations of more pronounced genome downsizing with higher ploidy [30, 45, 55–57]. Recent work [57] has demonstrated erosion of low copy-number repetitive DNA in allopolyploids, sometimes counteracted by expansion of a few repeat types. Age and genomic similarity of the parental genome donors of the polyploids play a role in the extent of genome size change with polyploidy [56] and a deeper understanding of the likely hybridogenic origin of some of the *Tanacetum* polyploids studied would allow more robust hypotheses on the balancing genomic processes these taxa may have undergone.

### Small genome size and invasiveness

*Tanacetum parthenium* appears listed in several countries as an invasive weed [58, 59]. Its genome size was the smallest obtained in our study (three populations were analysed whose mean was 2C = 4.12 pg). This is consistent with previous findings [60], which detected that many weeds (including those in family Asteraceae) had smaller amounts of DNA than closely related (non-weedy) species. This relationship is supported by recent work [61, 62]. The other species with small genome sizes in our sample (*T. parthenifolium* and *T. persicum*) have not, however, been recorded as weeds. Therefore a small genome size (particularly, smaller than that of closely related species) is a necessary but not sufficient condition for a plant to become a weed. A recent review [63] concluded that invasive species were characterised by small and very small genomes, yet this conclusion may be biased by the general trend of land plants to small genome sizes as a whole [42].

### Intraspecific instability and massive amplification of GC-rich DNA occur in several *Tanacetum* species

We found that ribosomal DNA is always CMA+ in *Tanacetum* (see Discussion on rDNA loci below), common to other studies [45, 64, 65] although exceptions are found [66]. For most of the studied populations, the number of CMA+ bands significantly exceeded that of rDNA signals and there was no apparent relationship with ploidy or with genome size (Table 1). The number of CMA+ bands is neither stable within a species nor within a population. The presence of odd and of non-homologous signals was occasionally observed, for example in *T. aureum* and in *T. oligocephalum* (Table 1), where a single chromosome with two CMA+ bands at each end was observed instead of the two identical chromosomes expected. Odd ploidy species, such as *T. fisherae* (5x) and *T. kotschyi* (3x), were particularly labile with respect to the number of CMA+ bands. However, the greatest variability in number of CMA+ bands corresponded to the diploid *T. balsamita*, in which sevendifferent numbers of signals were found (Table 1 and Fig. 3c). Such instability in the number of GC-rich bands was unexpected and has seldom been reported. Only the highly variable CMA+ banding pattern previously found in *Citrus* L. and close genera [67] is similar to the variability found in *Tanacetum*, probably as a consequence of amplification or reduction in satellite sequences known to be particularly GC-rich [68]. It is possible that some as yet undescribed satellite DNA type, specific to *Tanacetum*, is in part responsible for these karyotype features.

Another characteristic of the CMA+ banding pattern in *Tanacetum* was the striking number of signals found in certain species, particularly in diploid taxa (Table 1, Fig. 3a, 3c, 3i, 3k). This contrasts with previous work on genus *Artemisia* [69, 70], in which a large number of CMA+ bands was only detected in some polyploids, while the only CMA+ bands in diploids were those exactly corresponding to rDNA loci. In other Asteraceae genera, such as *Cheirolophus* Cass., a large number of CMA+ bands was also reported, mostly coincidental with 35S rDNA signals [71]; this was also the case for *Filifolium* [72]. In *Centaurea* L. [73] the number of CMA+ bands was the same as or smaller than the number of 35S rDNA signals, while in some *Xeranthemum* L. [74], *Galinsoga* Ruiz & Pav. and *Chaptalia* Vent. [75], few additional GC-rich bands were observed.

While most bands are in terminal position, pericentromeric GC-rich heterochromatin was detected in several species, some of them closely related, such as *T. polycephalum*, *T. aureum* and *T. canescens* DC. on one hand (Table 1), and *T. fisherae* (Fig. 2j), *T. kotschyi* (Fig. 2d), *T. tenuisectum* Sch.Bip. and *T. joharchii* (Fig. 3k) on the other. In fact, in *Arabidopsis thaliana* (L.) Heynh., centromeres are one of the most GC-rich genomic regions [76]. Differences in total GC% among eukaryotes are largely driven by the composition of non-coding DNA of which retrotransposons are the most abundant (for example, LTR Huck elements contain more than 60 % GC, [77]). Possibly, some centromere-specific LTR could have undergone amplification in these closely related *Tanacetum* genomes.

What can this fluctuating distribution of CMA+ bands mean, and what are the implications? It is feasible that a specific satellite and/or retroelement family may be expanded or contracted in *Tanacetum* genomes. Although the number and the distribution of CMA+ bands are thought to be relatively constant features of plant karyotypes [24, 70], our results strongly argue against this view, since variability was found even within a population. In addition, there were few evident ecological or geographic patterns in *Tanacetum*, that is, few significant relationships were found between the number or variability of GC-rich signals and geographical distribution, weedy behaviour, or soil features. The only significant association is with altitude: *Tanacetum* species living at higher altitudes tend to present more GC-rich DNA. In line with this hypothesis, [78] found a large number of heterochromatic bands (both GC- and AT-rich) in species from the Asteraceae genus *Myopordon* Boiss. inhabiting high mountain areas. These authors related the development of such heterochromatic bands in terminal regions with an adaptation to protect telomere function from UV radiation, a major genome-damaging agent, particularly in high mountains. Heterochromatin expansion in terminal regions (as in *Tanacetum*) has also been suggested to enhance chromosomal pairing during cell division [79].

### Genomic organisation of rDNA and typical distribution pattern of *Tanacetum*

Our cytogenetic study confirmed that both the 5S and the 35S rRNA genes are co-localised (L-type arrangement) in all chromosomes. Such organisation was found in *Artemisia* for the first time in higher plants [36], and subsequently inferred for at least 25 % of Asteraceae species [25]. In the latter study, Southern blot hybridisation was performed on a sample of *T. parthenium*, and the profile obtained also suggested L-type organisation for its rDNA. Prior to our study, the only evidence of this particular rDNA organisation directly in chromosomes was from *T. achilleifolium* and *T. parthenium* [35]. Curiously, these authors found one unlinked 5S locus additional to two regular L-type loci in *T. achilleifolium*, while *T. parthenium* showed L-type arrangement in all loci. Within the sample studied we could not find a single species with unhomogenised rDNA (i.e. that both kinds of rDNA arrangement, linked and separated, were present in the same species), since both rDNA probes invariably overlapped in all loci. Nevertheless, possible incomplete homogenisation of rRNA genes may also be present in other close genera such as *Achillea* and *Chrysanthemum* L. [72, 80]. Besides, in some metaphases decondensed rDNA signals are detected. These probably correspond to active nucleolar organizer regions (NORs), i.e. rDNA that is being actively transcribed, visible in *T. balsamita* (Fig. 3d, one signal) and in *T. joharchi* (Fig. 3l, two signals). Decondensed rDNA, however, is not always detected during metaphase.

### Unexpected variation in number of rDNA loci

The number of rDNA signals was always smaller and less variable than that of CMA+ bands, as found previously in other closely related species (in *Artemisia*, [45, 70]) and even in other families (genus *Ipomoea* from Convolvulaceae, [81]). In particular, the most common number of rDNA loci at the diploid (with two to three loci) and tetraploid (with five to six loci) levels was relatively constant and consistent with previous data for *Tanacetum* [35, 82] or for the closely related genera *Matricaria* and *Tripleurospermum* [25]. However, taxa with odd, higher ploidy or aneuploid levels often displayed higher intraspecific polymorphism in the number of signals. Of these, the hypoaneuploid population of *T. polycephalum* var. *argyrophyllum* was particularly striking, since metaphases with 10, 11, 12, 13, 14 and 15 rDNA signals were observed; the hypoaneuploid *T. fisherae* (2n = 5x = 44) showed a similar condition (Table 1). Thus, processes of hypoaneuploidy could affect genomic stability producing this variation in number of loci.

Although it would be expected that the number of signals remain relatively constant for a given species, cases of intraspecific polymorphism in the number of signals are increasingly reported. As for *Tanacetum*, diversity in the number of rDNA signals for a given species has been found in *Fragaria vesca* L. [26] and in *Phaseolus vulgaris* L. [83], for example. However, what is exceptional in *Tanacetum* is that these polymorphisms happen even at the population level and, albeit very rarely, sometimes within the same individual. All this, together with the unexceptional situation of odd numbers of signals in many taxa (which otherwise is rare) illustrates how dynamic *Tanacetum* genomes are.

Given these fluctuations, the constantly terminal position of rDNA signals in all the species studied could be considered surprising. However, this is so in most plants: [84] argued that there seems to be a strong positive selection favouring the location of 35S rDNA at chromosome ends, probably as a result of homologous recombination constraints.

As with the number of CMA+ bands, there was no global reduction in the number of signals per haploid genome with increasing ploidy. Similarly, the number of rDNA loci did not show any apparent relationship with genome size.

Our analyses have allowed us to distinguish some interesting relationships between several of the traits studied. As others have found [85, 86] morphological data regarding pollen size are tightly linked with genome size in *Tanacetum,* i.e. pollen size reflects genome size in this genus. In addition, species of *Tanacetum* with solitary capitula have smaller genome sizes than those with capitula organised in complex inflorescences. It is known that sometimes polyploids tend to present larger reproductive organs and more flowers per inflorescence than their diploid relatives [87], but few studies have approached the relationship of genome size or polyploidy with natural patterns, such as inflorescence architecture [88]. Suggested that the shift in inflorescence phyllotaxis from spiral to distichous would have occurred at the same time as the expansion of genome size characterising several groups of grasses [89], though admitting no clear reason why genome size as such should affect inflorescence architecture.

In addition, the reconstruction of ancestral cytogenetic traits brings evidence that these characters have followed increases and decreases during evolution in *Tanacetum* (Fig. 4). In general, it seems that genome size and the number of rDNA loci have increased, while the number of CMA+ bands has decreased in most present taxa. Few studies have specifically approached the evolution of cytogenetic traits within a temporal and phylogenetic perspective and, while events favouring increase in genome size and number of rDNA signals during evolution have been detected [56], there is no discernible pattern in the direction of these changes. For example, [90] found a decrease in number of rDNA loci during the evolution of *Hypochaeris* L. The overall decrease of GC-rich DNA could also respond to depletion of certain repeated DNA sequences during evolution in *Tanacetum*.

## Conclusions

This work is the first extensive cytogenetic report on *Tanacetum* species. We have confirmed linkage of both rDNAs in all chromosomal loci. *Tanacetum* stands out as variable, particularly in the number of rDNA sites and CMA+ bands. These vary widely even within a given population. In particular, aneuploid and odd ploidy taxa appear more unstable. The observed intrapopulation differences are likely a reflection of genomic differentiation which could complement further population biology studies. Besides, the number of GC-rich DNA bands found in certain species is striking and deserves more study. A possible cause is the amplification of repeat families or TEs in these species compared to others showing utterly different profiles. Polyploidy and aneuploidy are important evolutionary forces in this genus. Several of the studied populations present spontaneous mixed ploidy, another sign of its current genomic dynamism.

It is difficult to set general patterns in the evolution of genome size, number of rDNA loci or heterochromatin in plants. Yet, studies such as ours contribute to the knowledge of these cytogenetic features at a larger scale. Finally, the particularly labile cytogenetic scenario observed in *Tanacetum* is uncommon and has been seldom reported. Both chromosomal markers (rDNA loci and GC-rich bands) tend to be relatively constant at the species level, a feature that has allowed their use in biosystematics. Still, even at the population level, these traits can be variable in *Tanacetum* and this variation is better understood considering evolutionary relationships between species.

## Methods

### Plant materials

Seeds of 38 populations of *Tanacetum* species were collected from the wild for molecular cytogenetics and genome size assessments (Table 1). Specimen vouchers of the studied materials have been deposited at the Medicinal Plants and Drug Research Institute Herbarium (MPH) of the Shahid Beheshti University, Tehran.

### Chromosome preparations

Root tip meristems were obtained by germinating achenes on moist filter paper in Petri dishes at room temperature in the dark. They were pre-treated with 2 mM 8-hydroxyquinoline at room temperature for 3–3.5 h. Subsequently, the material was fixed in 3:1 v/v absolute ethanol:glacial acetic acid and stored at 4 °C for 24 h, and then stored in 70 % ethanol at 4 °C until use. For fluorochrome banding and fluorescence *in situ* hybridisation (FISH), the chromosome spreads were obtained using the air-drying technique of [91], with modifications. Fixed root tips were washed three times in distilled water with shaking and later in citrate buffer (0.01 M citric acid-sodium citrate, pH 4.6) for 30 min, excised and incubated for 20–35 min at 37 °C in an enzymatic mixture [4 % cellulase Onozuka R10 (Yakult Honsha), 1 % pectolyase Y23 (Sigma) and 4 % hemicellulase (Sigma)]. Digested root tips were placed on a slide, excess enzymatic solution was removed and protoplasts were obtained by applying gentle pressure in a drop of 45 % acetic acid. The metaphase plates were evaluated using a phase contrast microscope and slides were frozen for at least 24 h at -80 °C. Later, the coverslip was quickly removed, the slide rinsed with absolute ethanol and then air dried for at least two days protected from dust.

### Fluorochrome banding

In order to reveal GC-rich bands, the chromosomes were stained with the fluorochrome chromomycin A_3_ (CMA), according to [24, 92] with slight modifications. The slides were incubated in McIlvaine buffer pH 7, MgSO_4_ (0.1 g/L in McIlvaine buffer, pH 7) for 15 min, stained with CMA_3_ (0.2 mg/ml in McIlvaine buffer pH 7 MgSO_4_) for 90 min in the dark, rinsed in McIlvaine buffer pH 7, and counterstained with methyl green (0.1 % in McIlvaine buffer pH 5.5) for 10 min; rinsed in McIlvaine buffer pH 5.5, dried briefly at room temperature, also in the dark, and mounted in two small drops of Citifluor AF1 (glycerol/PBS solution).

### Labelling of rDNA probes and FISH

For hybridisation experiments we mostly used the same slides as for fluorochrome banding with CMA after destaining with fixative, dehydration through an ethanol series (70 %, 90 % and 100 %) and drying for two days. The probe used for 35S rDNA localisation was a plasmid carrying a 2.5 kb insert of 26S rRNA gene from *Lycopersicum esculentum* Mill. labelled with Cy3 (Jena Biosciences) using the Nick Translation Mix (Roche). The 5S rDNA probe was an approximately 0.7 kb-long trimer of 5S rRNA genes from *Artemisia tridentata* Nutt., labelled with Green dUTP using the Nick Translation Mix (Abbott Molecular). This probe contained three units of the 5S rRNA gene (120 bp) and the non-coding intergenic spacers (about 290 bp). Both probes have been used following previous research [25, 65]. FISH was carried out according to [24] with slight modifications. Slides were incubated in 100 μg/ml DNase-free RNase in 2 × SSC (0.03 M sodium citrate and 0.3 M sodium chloride) for 1 h at 37 °C, washed in 2xSSC three times for 5 min with slow shaking, rinsed in 0.01 N HCl for 2 min and incubated in pepsin (0.1 mg/ml in 0.01 N HCl) for 15 min at 37 °C, washed in 2xSSC for 5 min twice, dehydrated in an ethanol series (70 %, 90 % and 100 %, for 3 min in each) and air dried. The probe hybridisation mixture contained 25–100 ng/μl rDNA probes, formamide, 50 % (w/v) dextran sulphate, and 20 × SSC. This was denatured at 75 °C for 10 min and chilled on ice for 5 min. A volume of 30 μl was loaded onto slides and covered with plastic coverslips. The preparations were denatured at 75 °C for 10 min and transferred at 55 °C for 5 min. Hybridisation was carried out for more than 18 h at 37 °C in a humidified chamber. Following hybridisation, the slides were washed with shaking in 2 × SSC, 0.1 × SSC and 2 × SSC at 42 °C for 5 min twice each, and then once in 2 × SSC for 5 min, once in 4 × SSCT for 7 min, briefly rinsed in 1 × PBS and dried.

Samples were counterstained with Vectashield (Vector Laboratories, Inc., Burlingame, CA, USA), a mounting medium containing 500 ng/μl of 4’,6-diamidino-2-phenylindole (DAPI). The fluorescence signals were analysed and photographed using a digital camera (AxioCam HRm, Zeiss) coupled to a Zeiss Axioplan microscope; images were analysed with Axiovision HR Rev3, version 4.8 (Zeiss) and processed for colour balance, contrast and brightness uniformity in Adobe Photoshop. A minimum of 10 metaphase plates per population were analysed. Graphics were assembled with PowerPoint 2010 (Microsoft). The data were submitted to the Plant rDNA database, a database compiling information on rDNA signal number, position and organisation [93, 94].

### Flow cytometric measurements

For flow cytometric measurements of leaf tissue, seedlings were obtained from seeds grown in pots in the greenhouse of the Faculty of Pharmacy, University of Barcelona. Five individuals per population of the different *Tanacetum* species were studied, and of these, two samples of each were individually processed. *Petunia hybrida* Vilm. ‘PxPc6’ (2C = 2.85 pg), *Pisum sativum* L. ‘Express Long’ (2C = 8.37 pg) and *Triticum aestivum* L. ‘Chinese Spring’ (2C = 30.9 pg) from [95] were used as the internal standards. Fresh leaf tissue for the standard and the target species were chopped up together in 600 μl of LB01 buffer (8 % Triton X-100; [96]) supplemented with 100 μg/ml ribonuclease A (RNase A, Boehringer, Meylan, France) and stained with 36 μl of 1 mg/ml propidium iodide (Sigma-Aldrich, Alcobendas, Madrid, 60 μg/ml) to a final concentration of 60 μg/ml, and kept on ice for 20 min. The fluorescence measurements were performed using an Epics XL flow cytometer (Coulter Corporation, Miami, FL, USA) at the Centres Científics i Tecnològics, University of Barcelona. More details about the method are in [55]. The data have been submitted to the GSAD (Genome Size in Asteraceae Database) [97, 98].

### Phylogenetic analyses and reconstruction of character evolution

The nuclear ITS1 + ITS2 and chloroplast trnH-psbA sequences (listed in Additional file 1) were edited by BioEdit v. 7.1.3.0 [99] followed by manual adjustment. *Artemisia* taxa were considered as outgroups [3]. All taxa used for the phylogenetic analysis were diploid in order to avoid the effect of polyploidy in the estimated nuclear DNA contents, number of rDNA sites or GC-rich bands. Bayesian phylogenetic analysis was performed in MrBayes 3.1.2 [100] using a SYM + G model determined from jModeltest v. 2.1.3 [101] under the Akaike information criterion (AIC; [102]), to ascertain phylogenetic relationships. The Markov chain Monte Carlo (MCMC) sampling approach was used to calculate posterior probabilities (PPs). Four consecutive MCMC computations were run for 2,000,000 generations, with tree sampling every 100 generations. Data from the first 1000 generations were discarded as the burn-in period. PPs were estimated through the construction of a 50 % majority-rule consensus tree.

The ancestral character reconstructions (genome size, number of rDNA sites and number of CMA+ bands) were conducted using unordered maximum parsimony as implemented for continuous and meristic characters in Mesquite v. 3.02 software [103] using the 50 % majority-rule consensus tree resulting from the Bayesian inference analysis as the input tree file. The output trees were edited with Mesquite v. 3.02.

### Statistical analyses

Analyses of regression, one-way ANOVA, *X*^2^, Shapiro-Wilk test for normality and Barlett’s test for equality of variances were performed with RStudio, v.0.98.1078. In addition, the phylogenetic generalised least squares (PGLS) algorithm as implemented in the *nlme* package for R (Version 3.1-118) was used to analyse variation of genome size, number of rDNA sites and number of CMA+ bands in a phylogenetic context. Data on genome size and ribosomal DNA loci for the complementary and outgroup species were extracted from the Plant rDNA database [93].

### Availability of supporting data

The data sets supporting the results of this article are available in the TreeBase repository, ID 17805 and http://purl.org/phylo/treebase/phylows/study/TB2:S17805 [104].

## Additional file

Additional file 1:
**Accessions downloaded from GenBank.** Species names and accession numbers of *Artemisia* and *Tanacetum* ITS1 + ITS2 and trnH-psbA sequences.

## Abbreviations

1Cx: Monoploid Genome Size
2C: Holoploid Genome Size
CMA: Chromomycin A_3_
FISH: Fluorescent *in situ* Hybridisation
NOR: Nucleolar Organizer Region
PGLS: Phylogenetic Generalised Least Squares
rDNA: Ribosomal DNA (or ribosomal RNA genes)
rRNA: Ribosomal RNA
TKL: Total Karyotype Length
