# Supplementary material for: The striking and unexpected cytogenetic diversity of genus Tanacetum L. (Asteraceae): a cytometric and fluorescent in situ hybridisation study of Iranian taxa
Source: BMC Plant Biol. 2015 Jul 8;15:174. doi: 10.1186/s12870-015-0564-8 (PMC4494159; doi:10.1186/s12870-015-0564-8)
Supplement: Additional file 1: — Accessions downloaded from GenBank. Species names and accession numbers of Artemisia and Tanacetum ITS1 + ITS2 and trnH-psbA sequences. [file 12870_2015_564_MOESM1_ESM.docx]

**Additional file 1** Accessions downloaded from GenBank.

| Species | ITS1+ ITS2 Accession | trnH-psbA Accession |
| --- | --- | --- |
| *Tanacetum polycephalum* ssp. *argyrophyllum* | [AB683305.1](http://www.ncbi.nlm.nih.gov/nuccore/AB683305.1) | [AB683418.1](http://www.ncbi.nlm.nih.gov/nuccore/AB683418.1) |
| *Tanacetum aureum** | [AB683275.1](http://www.ncbi.nlm.nih.gov/nuccore/AB683275) | [AB683387.1](http://www.ncbi.nlm.nih.gov/nuccore/AB683387.1) |
| *Tanacetum sonboliI* | [AB683323.1](http://www.ncbi.nlm.nih.gov/nuccore/AB683323.1) | [AB683431.1](http://www.ncbi.nlm.nih.gov/nuccore/AB683431.1) |
| *Tanacetum vulgare* | [EF577323.1](http://www.ncbi.nlm.nih.gov/nuccore/EF577323.1) | [AB683438.1](http://www.ncbi.nlm.nih.gov/nuccore/AB683438.1) |
| *Tanacetum hololeucum* | [AB683289.1](http://www.ncbi.nlm.nih.gov/nuccore/AB683289.1) | [AB683402.1](http://www.ncbi.nlm.nih.gov/nuccore/AB683402.1) |
| *Tanacetum pinnatum* | [AB683304.1](http://www.ncbi.nlm.nih.gov/nuccore/AB683304.1) | [AB683416.1](http://www.ncbi.nlm.nih.gov/nuccore/AB683416.1) |
| *Tanacetum budjnurdense* | [AB683274.1](http://www.ncbi.nlm.nih.gov/nuccore/AB683274.1) | [AB683386.1](http://www.ncbi.nlm.nih.gov/nuccore/AB683386.1) |
| *Tanacetum parthenium* | [FN823080.1](http://www.ncbi.nlm.nih.gov/nuccore/FN823080.1) | [AB683415.1](http://www.ncbi.nlm.nih.gov/nuccore/AB683415.1) |
| *Tanacetum parthenifolium* | [AB683303.1](http://www.ncbi.nlm.nih.gov/nuccore/AB683303.1) | [AB683413.1](http://www.ncbi.nlm.nih.gov/nuccore/AB683413.1) |
| *Tanacetum tenuisectum* | [AB523747.1](http://www.ncbi.nlm.nih.gov/nuccore/AB523747.1) | [AB683434.1](http://www.ncbi.nlm.nih.gov/nuccore/AB683434.1) |
| *Artemisia vulgaris* | [JX051678.1](http://www.ncbi.nlm.nih.gov/nuccore/JX051678.1) | [JX073849.1](http://www.ncbi.nlm.nih.gov/nuccore/JX073849.1) |
| *Artemisia absinthium* | [JX051763.1](http://www.ncbi.nlm.nih.gov/nuccore/JX051763.1) | [JX073911.1](http://www.ncbi.nlm.nih.gov/nuccore/JX073911.1) |

(*) Previous name: *Tanacetum chiliopyllum* var. *chilliophyllum*
